# Piezo type mechanosensitive ion channel component 1 functions as a regulator of the cell fate determination of mesenchymal stem cells

**DOI:** 10.1038/s41598-017-18089-0

**Published:** 2017-12-18

**Authors:** Asuna Sugimoto, Aya Miyazaki, Keita Kawarabayashi, Masayuki Shono, Yuki Akazawa, Tomokazu Hasegawa, Kimiko Ueda-Yamaguchi, Takamasa Kitamura, Keigo Yoshizaki, Satoshi Fukumoto, Tsutomu Iwamoto

**Affiliations:** 1 Department of Pediatric Dentistry, Institute of Biomedical Sciences, Tokushima University Graduate School, Tokushima, 770-8504 Japan; 2 Support Center for Advanced Medical Sciences, Institute of Biomedical Sciences, Tokushima University Graduate School, Tokushima, 770-8504 Japan; 3 Section of Orthodontics and Dentofacial Orthopedics, Division of Oral Health, Growth and Development, Faculty of Dental Science, Kyushu University, Fukuoka, 812-8582 Japan; 4 Division of Pediatric Dentistry, Department of Oral Health and Development Sciences, Tohoku University Graduate School of Dentistry, Sendai, 980-8575 Japan

## Abstract

The extracellular environment regulates the dynamic behaviors of cells. However, the effects of hydrostatic pressure (HP) on cell fate determination of mesenchymal stem cells (MSCs) are not clearly understood. Here, we established a cell culture chamber to control HP. Using this system, we found that the promotion of osteogenic differentiation by HP is depend on bone morphogenetic protein 2 (BMP2) expression regulated by Piezo type mechanosensitive ion channel component 1 (PIEZO1) in MSCs. The PIEZO1 was expressed and induced after HP loading in primary MSCs and MSC lines, UE7T-13 and SDP11. HP and Yoda1, an activator of PIEZO1, promoted *BMP2* expression and osteoblast differentiation, whereas inhibits adipocyte differentiation. Conversely, *PIEZO1* inhibition reduced osteoblast differentiation and *BMP2* expression. Furthermore, Blocking of BMP2 function by noggin inhibits HP induced osteogenic maker genes expression. In addition, in an *in vivo* model of medaka with HP loading, HP promoted caudal fin ray development whereas inhibition of piezo1 using GsMTx4 suppressed its development. Thus, our results suggested that PIEZO1 is responsible for HP and could functions as a factor for cell fate determination of MSCs by regulating BMP2 expression.

## Introduction

Osteoblast lineage cells and marrow adipocytes originate from a common progenitor cells in the bone marrow-derived mesenchymal stem cells (BMSCs). Numerous studies have indicated that adipogenesis-induction factors inhibit osteoblastogenesis, whereas osteoblastogenesis-induction factors block adipogenesis^1^, indicating a reciprocal relationship between osteoblastogenesis and adipogenesis^2^. Furthermore, in aging and osteoporosis, an enhanced adipogenesis is observed relative to osteoblastogenesis in the bone marrow, which correlates with reduced trabecular bone mass^3^. Hence, elucidation of the molecular mechanisms responsible for controlling the balance between osteoblastogenesis and adipogenesis is of substantial importance to improve the treatment strategies for skeletal disease.

The self-renewal and cell fate decisions of MSCs are extremely sensitive to changes in the extracellular environment and related factors, including extracellular matrix stiffness^4^, cell culture medium^5^, O_2_ concentration^6^, three-dimensional scaffolds^7^, and mechanical stress. In particular, mechanical stress constitutes an essential factor for bone homeostasis and osteogenesis in skeletal tissue. The situation of lacking a mechanical force such as the long-term bedridden and microgravity environment decrease bone mass^8,9^. Alternatively, increasing loading stimuli, e.g., through exercise and vigorous activities, enhance bone mass^10^. To prevent skeletal fragility, various growth factors, hormones, and chemical compounds are administered, promoting osteoblast activity or inhibiting osteoclast activity; however, in the absence of external pressure load from the external environment, reduction of bone mass cannot be suppressed, even if appropriate medicines are used. Therefore, understanding the molecular mechanisms underlying the cellular response to mechanical force may lead to the novel therapeutic strategies.

Osteoblastogenesis and bone formation are mediated by several cytokines, including bone morphogenetic proteins (BMP), transforming growth factor β, Wnt, and hedgehog^11–15^. Among these factors, BMP2 is a potent growth factors that plays a critical role in osteoblast differentiation of MSCs and osteoprogenitor cells *in vitro* and *in vivo* ^11,15^. Recombinant human BMP2 comprises the highly osteo-inductive growth factor used for bone regeneration and repair as approved by the US Food and Drug Administration (FDA)^16^. Moreover, during the process of osteogenesis from MSCs, two master transcription factors, *RUNX2* (also termed *CBFA1*) and *Osterix* (*OSX*) are required for osteoblast differentiation^17,18^. Runx2 directs MSCs into an osteoblastic lineage, but inhibits differentiation into the adipocytic and chondrocytic lineages^19^. After differentiating into pre-osteoblasts, cells express Runx2 and Osx to regulate the expression of osteogenic genes, such as alkaline phosphatase (*ALP*) and type and collagen type I alpha 1 chain (*COL1A1*)^20^. The expression and function of both Runx2 and Osterix are regulated by BMP2^21,22^.

Piezo type mechanosensitive ion channel component (Piezo) 1 and Piezo2 have been identified as mechanosensitive cation channels^23^. Piezo1 is primarily expressed in nonsensory tissues and non-neuronal cells, such as the bladder, kidney, lung, endothelial cells, erythrocytes, periodontal ligament cells, and chondrocytes, whereas Piezo2 is predominantly expressed in sensory tissues, such as dorsal root ganglia (DRG) sensory neurons and Merkel cells^24^. Piezo ion channels function in a variety of physiological processes such as regulation of red blood cell volume and sensation of gentle touch^25^. Moreover, Piezo1-deficient mice exhibited disrupted vasculature and embryonic lethality at midgestation with defects in vascular remodelling, suggesting that Piezo1 plays a critical role in the control of vascular architecture and embryonic development^26^. Interestingly, it has been recently reported that Piezo1 could be associated with mechanosensitive lineage specification in neural stem cell^27^. Human *PIEZO1* is a causative gene for hereditary xerocytosis, a dominant disorder of erythrocyte dehydration with haemolytic anaemia^28^. Mutations in *PIEZO2* cause Gordon syndrome, Marden-Walker syndrome, and distal arthrogryposis type 5, characterized by muscular contracture and cleft palate^29^. However, the expression and function of mechanosensitive PIEZO ion channels in MSCs and osteoblasts have not yet been established.

Accordingly, in this study, we demonstrate for the first time that PIEZO1 functions as a receptor for HP in MSCs and promotes osteoblast differentiation, whereas inhibits adipocyte differentiation. Among mechanosensing receptors, *PIEZO1* is preferentially expressed in MSCs. HP activates ERK1/2 and p38 MAPK signalling through PIEZO1, followed by the induction of *BMP2* expression. Blocking of BMP2 function inhibited HP-induced osteogenic maker genes expression. Thus, our results suggest that PIEZO1 functions as the cell fate determination factor in MSCs by regulating the BMP2 expression. Our findings provide important insights into the role of PIEZO1 as a target for skeletal diseases.

## Results

### Optimum HP promotes osteogenesis, but inhibits adipogenesis in MSC lines

To analyse the response of mesenchymal stem cells (MSCs) to HP, we developed an original and airtight acrylic cell-culture chamber that can control HP with an extracellular gaseous phase in the range of 0 to 0.03 MPa.

**Figure 1:**
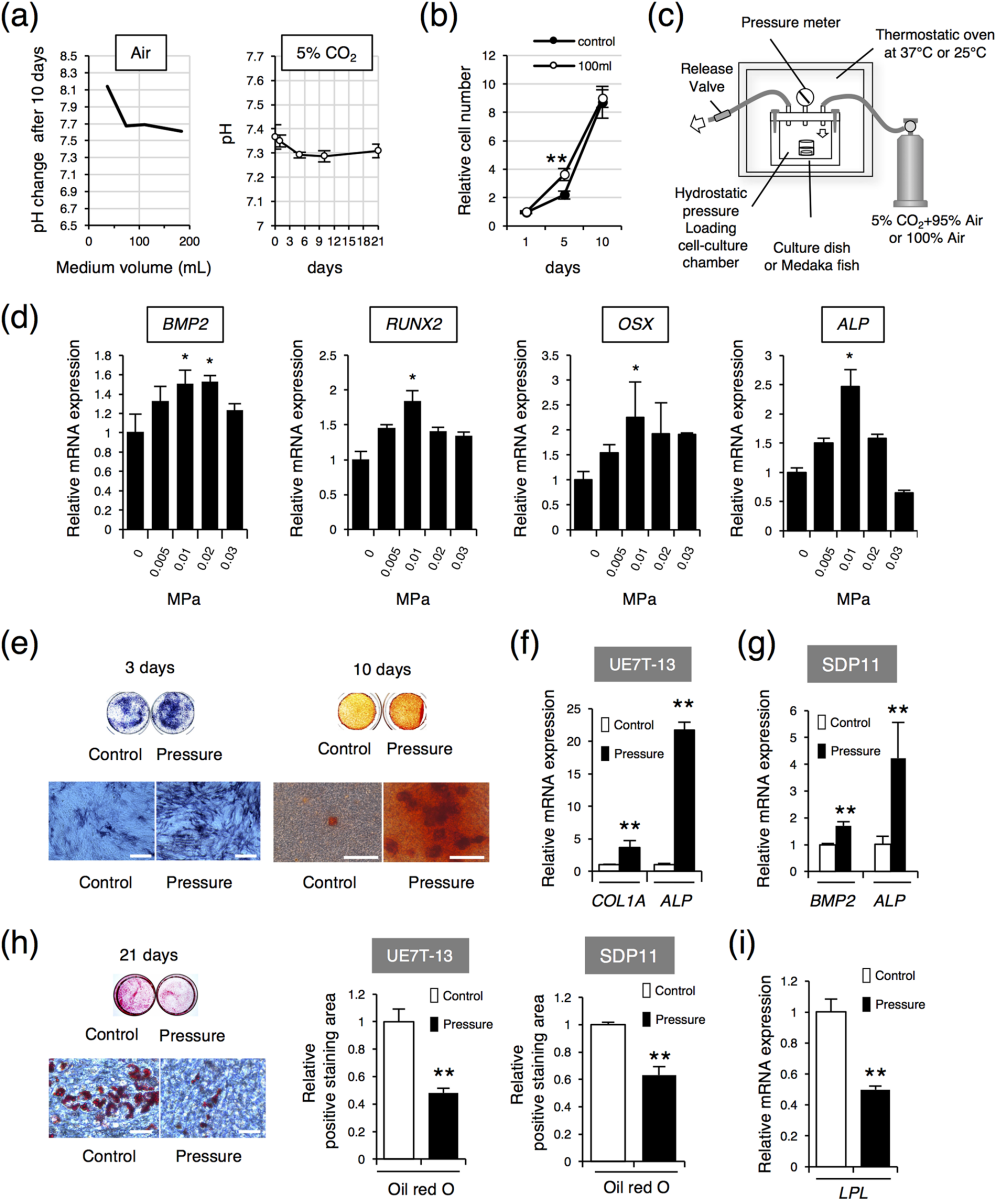
Hydrostatic pressure promotes osteoblastic differentiation and inhibits adipogenic differentiation in MSC lines. (**a**) Medium pH changes under atmospheric air or 5% CO_2_ at 37 °C after 10 days. UE7T-13 cells were cultured with different amounts of medium under atmospheric air (left). UE7T-13 cells were cultured with 100 mL medium under 5% CO_2_ (right). (**b**) Cell proliferation. UE7T-13 cells were seeded at 1 × 10^5^ cells in a 35-mm culture dishes. For the control, 2 mL cell medium was exchanged every 2 days. The experimental group was continuously cultured with 100 mL medium. Cell numbers were counted at indicated days. (**c**) Schematic diagram of the custom-made pressure chamber. (**d**) The UE7T-13 cells were cultured under different HP for 24 h, and osteoblast marker gene expression levels were examined by real-time RT-PCR. (**e**) ALP and Alizarin Red staining. UE7T-13 cells were cultured with osteogenic differentiation medium under 0.01 MPa HP loading. After 3 days of culture, ALP staining was performed (left, scale bar: 200 μm). After 10 days of osteogenic induction with 0.01 MPa HP loading, Alizarin Red staining was performed (right, scale bar: 300 μm). (**f)** Osteoblast marker genes expression in UE7T-13. Total RNA was extracted after 10 days of osteogenic induction with 0.01 MPa HP loading and analysed by real-time RT-PCR. (**g**) Osteoblast marker genes expression in SDP11 cells. Total RNA was extracted after 24 h of osteogenic induction with 0.01 MPa HP loading and analysed by real-time RT-PCR. (**h**) Oil Red O staining. UE7T-13 cells were cultured with adipogenic differentiation medium under the 0.01 MPa HP loading for 21 days, and Oil Red O staining was performed (scale bar: 100 μm). The Oil Red O positive area was quantified using ImageJ. (**i**) *LPL* gene expression. Total RNA was extracted after 3 days of adipogenic induction with 0.01 MPa HP loading and analysed by real-time RT-PCR. All Data were pooled from three independent experiments, and error bars indicate standard deviations. Statistical analysis was performed using analysis of variance (*p < 0.05, **p < 0.01).

First, we assessed the cell culture condition. To carry out cell culture under continuous HP loading with our chamber, the cells should be cultured without medium change. In general cell culture, medium change is necessary to avoid the accumulation of metabolic products such as lactic acid from the cultured cells and to prevent increased pH acidity. Therefore, the pH stability relative to the amount of medium in culture was measured. Human bone marrow-derived UE7T-13 cells were cultured in various amounts of media at 100% atmospheric conditions at 37 °C for 10 days. We found that the pH value of the medium was stable when more than 80 mL of medium was used for 10 days without medium changes under 100% atmospheric conditions (Fig. 1a). Therefore, we decided to use 100 mL of medium for continuous cell culture. Next, we observed the pH changes during UE7T-13 cells culture for 10 days with 100 mL of medium under 5% CO_2_ at 37 °C. After 10 days, the pH was stable at around 7.3 (Fig. 1a). Furthermore, after continuous culture in 100 mL medium without medium changes, there were no problems with cell proliferation (Fig. 1b). Hence, for subsequent experiments, cells were seeded on a 35-mm dish, placed in a 150-mL beaker, and incubated with 100 mL medium at 5% CO_2_ and 37 °C in the pressure chamber under HP loading. Control cells were cultured under the same conditions without HP.

Next, we sought to clarify the optimum HP for induction of osteoblastic cell lineage cells from MSCs using UE7T-13 cells (Fig. 1d) and assessed *BMP2*, *RUNX2*, *OSX*, and *ALP* expression by quantitative reverse transcription polymerase chain reaction (RT-PCR). The expression levels of these genes were increased in a force-dependent manner up to 0.01 MPa (Fig. 1d), suggesting that 0.01 MPa HP was optimum for induction of differentiation from MSCs into osteoblastic lineage cells.

**Figure 2:**
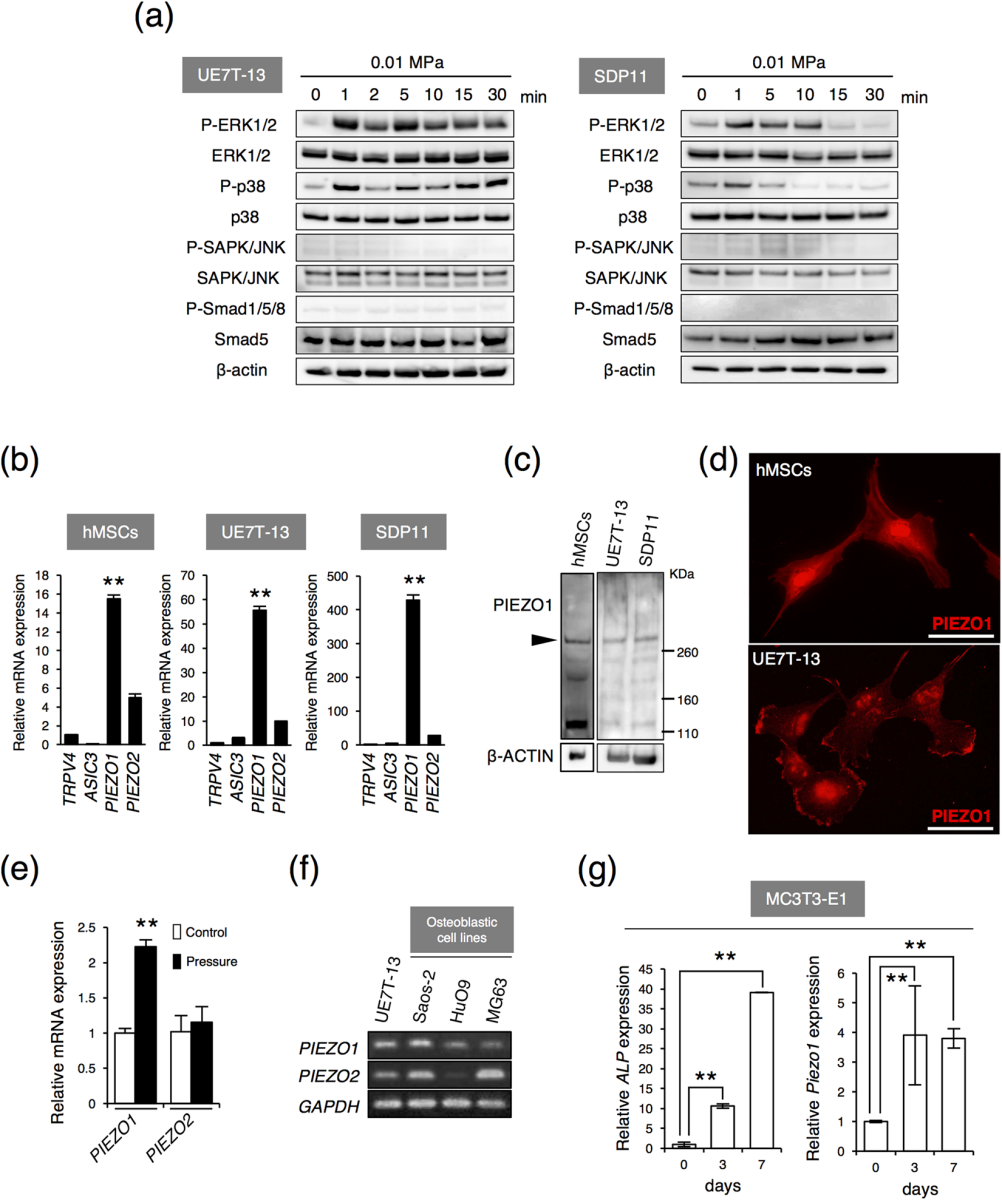
PIEZO1 expression in MSCs, MSC lines and osteoblast cell lines. (**a**) UE7T-13 (left) and SDP11 cells (right) were cultured under 0.01 MPa HP for the different time points indicated and then immunoblotted with specific antibodies. (**b**) Expression of mechanosensing receptors in hMSCs (left), UE7T-13 (middle), and SDP11 cells (right) were examined by real-time RT-PCR. Data were pooled from three independent experiments, and error bars indicate standard deviations. Statistical analysis was performed using analysis of variance (**p < 0.01). (**c**) PIEZO1 expression in hMSCs, UE7T-13, and SDP11 cells. PIEZO1 expression levels without induction were assessed by western blotting. β-Actin was used as an endogenous control. (**d**) Without induction, cellular localizations of PIEZO1 in hMSCs and UE7T-13 cells were determined by immunostaining. Scale bars: 20 μm. (**e**) *PIEZO1* expression after 0.01 MPa HP loading. UE7T-13 cells were cultured with osteogenic differentiation medium under 0.01 MPa HP loading. After 10 days of culture, *PIEZO1* and *PIEZO2* expression levels were examined by real-time RT-PCR. Data were pooled from three independent experiments and error bars indicate standard deviations. Statistical analysis was performed using analysis of variance (**p < 0.01). (**f**) *PIEZO1* expression in all osteogenic cell lines. *PIEZO1* and *PIEZO2* expression levels in human osteoblast cell lines were examined by RT-PCR. The PCR products were separated by 2% agarose gel electrophoresis and visualized by ethidium bromide staining. *GAPDH*, glyceraldehyde-3-phosphate dehydrogenase. (**g**) *Piezo1* expression in differentiating MC3T3-E1 cells. The MC3T3-E1 cells were cultured with osteogenic induction medium for 0, 3, and 7 days, and *ALP* and *PIEZO1* expression levels were examined by real-time RT-PCR. Data were pooled from three independent experiments and error bars indicate standard deviations. Statistical analysis was performed using analysis of variance (**p < 0.01).

We then examined the effects of 0.01 MPa HP on osteoblast and adipocyte differentiation. UE7T-13 cells and SDP11 cells were cultured under 0.01 MPa HP with osteogenic conditions. Alkaline phosphatase (ALP) staining was increased in HP-loaded UE7T-13 cells compared with that in control cells (Fig. 1e). Moreover, 0.01 MPa HP dramatically increased mineral nodule formation after 10 days of induction (Fig. 1e). Furthermore, the expression levels of osteogenic marker genes, i.e., *COL1A1* and *ALP* in UE7T-13 cells (Fig. 1f) and *BMP2* and *ALP* in SDP11 cells (Fig. 1g) were increased by 0.01 MPa HP compared with those in control cells after HP loading. Both UE7T-13 cells and SDP11 cells generated lipid vacuoles under the adipogenic condition; however, 0.01 MPa HP inhibited the lipid vacuole formation (Fig. 2h). In addition, the expression of lipoprotein lipase (*LPL*), a marker of adipocytes, was reduced by 0.01 MPa HP in UE7T-13 cells (Fig. 1i). These results suggested that 0.01 MPa HP promoted the differentiation of osteoblasts and inhibited the differentiation of adipocytes from MSCs. Thus, HP was strongly correlated with the cell fate decision of MSCs.

### Expressions of PIEZO1 in MSCs and MSC lines

Next, to clarify the initial cellular signaling associated with HP, cellular responses were analysed by western blotting. Mechanical stress activates mitogen activated protein kinases (MAPKs) in MSCs^30,31^. Therefore, we examined whether MAPK signaling was involved in the cellular responses to HP loading. Phosphorylation of extracellular signal-regulated kinase (ERK) 1/2 and p38 was observed at 1 min after stimulation of 0.01 MPa HP loading in UE7T-13 and SDP11 cells (Fig. 2a). However, c-Jun NH_2_-terminal kinase (JNK) was not activated by 0.01 MPa HP loading (Fig. 2a). In addition, 0.01 MPa HP strongly induced *BMP2* expression after 24 h loading (Fig. 1d and g). As BMP2 is known to activate the Smad signaling pathway, we tested whether this pathway was involved in cellular early response to HP. However, the phosphorylations of Smad1/5/8 were not observed within 30 min of HP loading (Fig. 2a). These results suggested that ERK1/2 and p38, but not Smad signaling, were involved in the early cellular response to 0.01 MPa HP.

**Figure 3:**
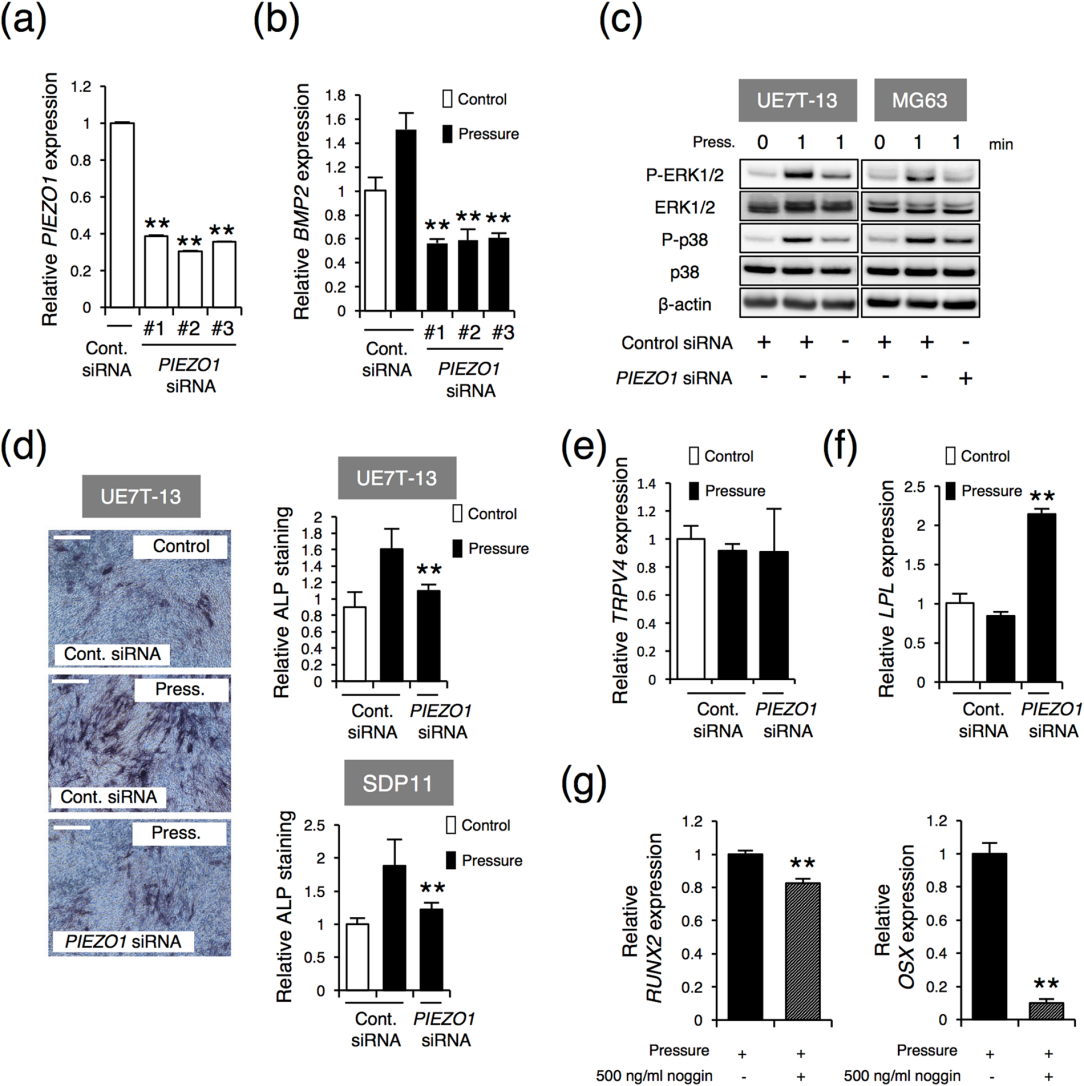
Inhibition of osteoblastic differentiation and promotion of adipogenic differentiation by PIEZO1 siRNA UE7T-13 cells were transfected with either control scrambled siRNA or three different targeted single siRNAs of *PIEZO1* (siRNA #1, siRNA #2, or siRNA #3). Total RNA was prepared from the transfected cells after 24 h and analysed by real-time RT-PCR (**a**,**b**,**e**). (**a**) Reduced expression of endogenous *PIEZO1*. (**b**) Reduced expression of *BMP2*. (**c**) Intracellular signalling. PIEZO1 siRNA-transfected UE7T-13 and MG63 cells were analysed by western blotting. (**d**) ALP activity staining. PIEZO1 siRNA was transfected into UE7T-13 and SDP11 cells, cells were cultured with HP loading for 3 days, and ALP activity staining was performed. The relative ALP-positive area was quantified using ImageJ. (**e**) *TRPV4* expression. (**f**) *LPL* expression. After transfection with *PIEZO1* siRNA into UE7T-13 cells, total RNA was extracted after 3 days of adipogenic induction cell culture with 0.01 MPa HP loading and analysed by real-time RT-PCR. (g) Inhibition of *RUNX2* and *OSX* expression. UE7T-13 cells were cultured with 500 ng/mL BMP noggin under 0.01 MPa HP loading for 24 h. Total RNA was prepared and analysed by real-time RT-PCR. Data were pooled from three independent experiments, and error bars indicate standard deviations. Statistical analysis was performed using analysis of variance (**p < 0.01).

Next, to identify the primary receptors to HP, we examined mechanosensing receptors expression levels in primary hMSCs and MSC lines, UE7T-13 and SDP11 cells. Real-time PCR revealed that those cells showed high expression of the mechanosensitive receptor *PIEZO1* (Fig. 2b). Western blot analysis demonstrated that PIEZO1 protein, with a predicted molecular mass of about 287 kDa, was also observed in those cells (Fig. 2c). Immunostaining with an anti-PIEZO1 antibody in hMSCs and UE7T-13 cells showed that PIEZO1 was expressed in the plasma membrane (Fig. 2d) and particularly the lamellipodia and filopodial tips (Fig. 2d). Moreover, *PIEZO1*, but not *PIEZO2* or *TRPV4*, was induced after 0.01 MPa HP loading (Figs 2e and 3e). These results suggest that the promotion of osteoblastic differentiation from MSCs by HP loading was correlated with PIEZO1 expression. Then, to further confirm that osteoblast differentiation is also actually correlated with PIEZO1, we have tested whether PIEZO1 is expressed in osteoblasts and in the process of osteoblast differentiation. Analysis of *PIEZO1* expression in human osteoblast cell lines showed that *PIEZO1* was expressed in Saos-2, HuO9, and MG63 cells. *PIEZO1* was expressed in all osteogenic lines (Fig. 2f). Furthermore, we found that *Piezo1* mRNA was induced in differentiating mouse osteoblastic MC3T3-E1 cells, an established experimental model for osteoblast differentiation (Fig. 2g). Thus, PIEZO1 appeared to act as the primary mechanosensing receptor for HP in MSCs, and correlated with osteoblast differentiation.

### PIEZO1 mediates the expression of *BMP2* to regulate osteoblast differentiation in MSC lines

To further elucidate the function of PIEZO1 in MSCs, we knocked down endogenous *PIEZO1* expression using *PIEZO1* siRNA. UE7T-13 cells were transfected with three different siRNAs targeting *PIEZO1*. Endogenous *PIEZO1* was reduced by approximately 65.0–72.4%, compared with that in control siRNA-transfected cells (Fig. 3a). The protein level was also reduced by *PIEZO1* siRNA (see Supplementary Fig. S1). We found that the induction level of *BMP2* by 0.01 MPa HP was dramatically inhibited by siRNA transfection (Fig. 3b).

Next, we examined the influence of endogenous *PIEZO1* knockdown on ERK1/2 and p38 phosphorylations (Fig. 2a). Notably, ERK1/2 and p38 phosphorylations were significantly reduced by *PIEZO1* siRNA transfection in UE7T-13 cells and the human osteoblast MG63 cell line (Fig. 3c). Furthermore, suppression of endogenous *PIEZO1* by si*PIEZO1* inhibited 0.01 MPa HP-induced ALP staining in both UE7T-13 and SDP11 cells (Fig. 3d). In *PIEZO1* siRNA-transfected UE7T-13 cells, the expression of *TRPV4* was not altered (Fig. 3e). In contrast, *LPL* expression was increased by *PIEZO1* siRNA transfection (Fig. 3f), suggesting that PIEZO1 was essential for HP-induced osteoblast differentiation, but not adipocyte differentiation in MSCs.

Then, to further confirm whether BMP2 induced by HP plays an important role in osteoblast differentiation from MSCs, a BMP2-specific antagonist, noggin, was administrated to the osteogenic induction culture with HP loading. Noggin significantly inhibited HP-induced *RUNX2* and *OSX* expressions (Fig. 3g). Collectively, these findings suggested that the BMP2 induced by PIEZO1 was important for the promotion of osteoblast differentiation and inhibition of adipocyte differentiation in MSCs.

### A PIEZO1 agonist promotes *BMP2* expression and osteoblast differentiation in MSC lines

**Figure 4:**
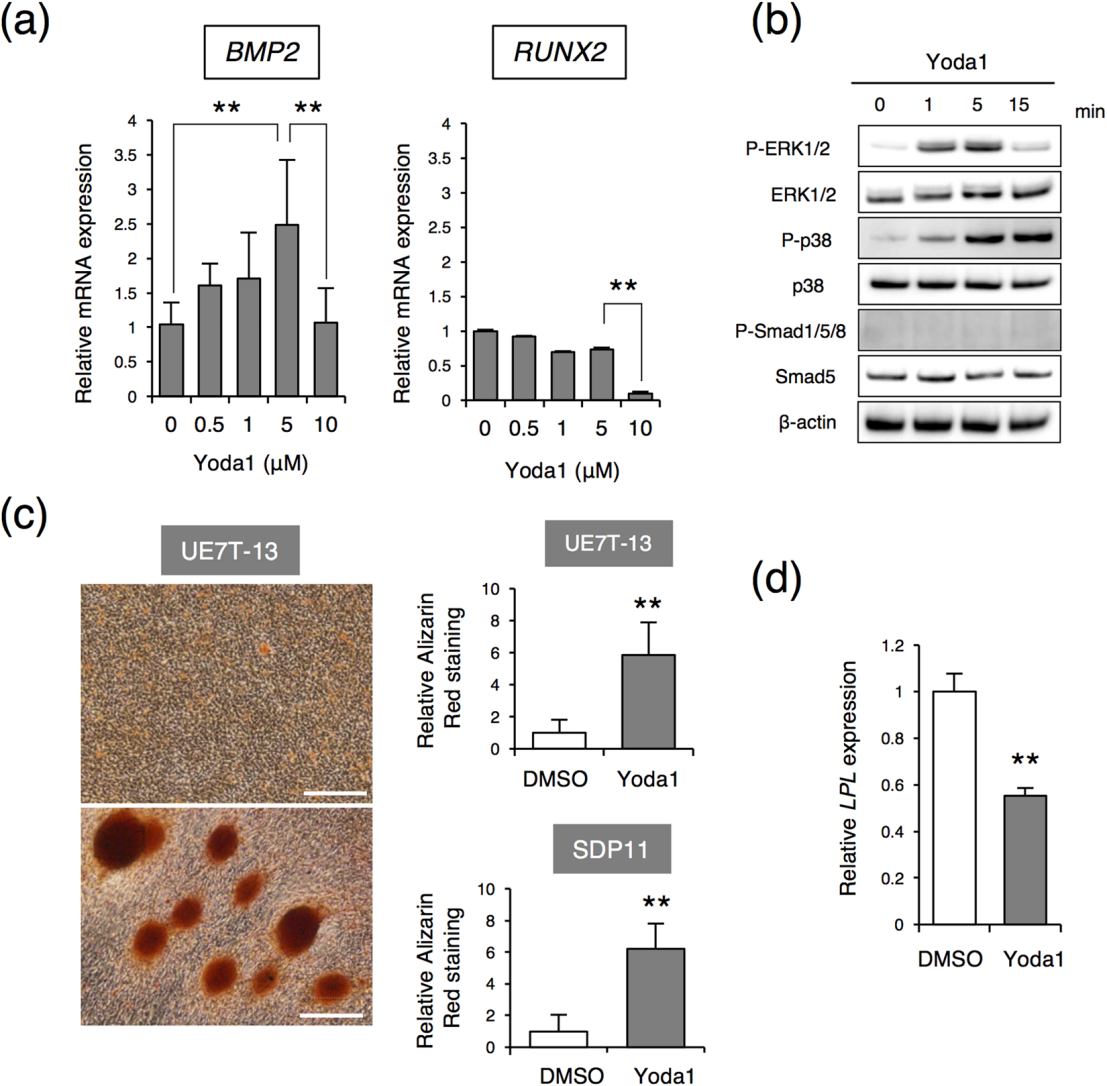
Promotion of osteoblastic differentiation and inhibition of adipogenic differentiation by Yoda1. (**a**) *BMP2* (left) and *RUNX2* (right) expression. Different concentrations of Yoda1 were applied to UE7T-13 cells for 24 h. Then, total RNA was prepared from the cells and analysed by real-time RT-PCR. (**b**) Intracellular signalling. Yoda1 treated UE7T-13 cells were analysed by western blotting with specific antibodies. (**c**) Alizarin Red staining. After 10 days of osteogenic induction cell culture with 5 μM Yoda1, Alizarin Red staining was performed with UE7T-13 cells and SDP11 cells. (**d**) *LPL* expression. Data were pooled from three independent experiments, and error bars indicate standard deviations. Statistical analysis was performed using analysis of variance (**p < 0.01).

We next examined whether PIEZO1 activation affected MSCs differentiation. We used a recently identified novel specific PIEZO1 agonist, Yoda1^32^. Notably, treatment of UE7T-13 cells with 5 μM Yoda1 for 24 h significantly induced *BMP2* but not *RUNX2* expression (Fig. 4a). Moreover, similar to the results of 0.01 MPa HP loading, Yoda1 promoted the activations of ERK1/2 and p38 (Fig. 4b). Phosphorylation of Smad was not observed within 15 min of Yoda1 treatment (Fig. 4b). These results indicated that ERK1/2 and p38 acted as an early response to Yoda1. Furthermore, to investigate whether PIEZO1 activation by Yoda1 promoted the formation of calcified nodules, UE7T-13 and SDP11 cells were cultured for 10 days with 5 μM Yoda1 added to osteo-inductive medium. Alizarin Red S staining revealed that Yoda1 promoted positive nodules formation in both UE7T-13 and SDP11 cells (Fig. 4c), indicating that osteoblast differentiation was enhanced in the presence of Yoda1. In contrast, Yoda1 suppressed *LPL* expression (Fig. 4d). Thus, similar to HP loading, Yoda1 induced *BMP2* expression and promoted osteoblast differentiation, but negatively regulated adipocyte differentiation in MSC lines. These results suggest that the differentiation of osteoblasts and adipocytes could be controlled by modulating PIEZO1 signaling without mechanical stimulation.

**Figure 5:**
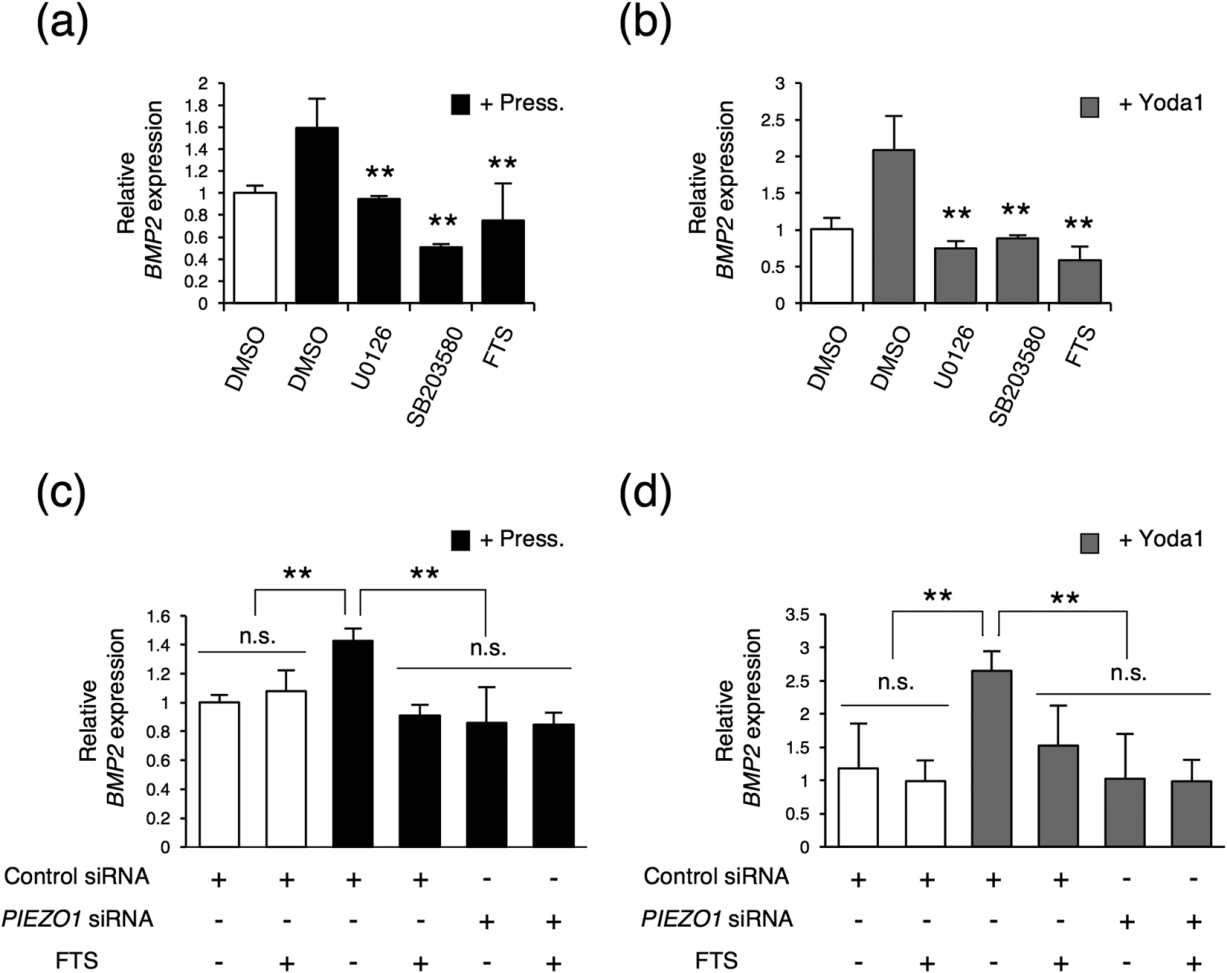
Inhibition of HP- and Yoda1-induced *BMP2* expression by MEK, p38, and Ras inhibitors. *BMP2* expression was analysed by real-time RT-PCR. UE7T-13 cells were cultured under 0.01 MPa HP (**a**) or 5 μM Yoda1 (**b**) in the presence of 10 μM U0126 (MEK inhibitor), 10 μM SB203580 (p38 inhibitor), or 10 μM FTS (Ras inhibitor) for 24 h. Total RNA was then extracted from the cells. On the other hand, UE7T-13 cells were transfected with control or PIEZO1 siRNA for 24 h. Then, the cells were cultured under 0.01 MPa HP (**c**) or 5 μM Yoda1 (**d**) with or without 10 μM FTS for 24 h. After that, total RNA was then extracted from the cells. Data were pooled from three independent experiments, and error bars indicate standard deviations. Statistical analysis was performed using analysis of variance (**p < 0.01).

Next, we examined whether activations of ERK1/2 and p38 modulated BMP2 expression. As shown in Fig. 5a and b, U0126 (a MEK inhibitor) and SB203580 (a p38 inhibitor) inhibited the *BMP2* expression induced by 0.01 MPa HP loading and Yoda1 treatment, suggesting that ERK1/2 and p38 signalling pathways were important for the BMP2 expression mediated by HP and PIEZO1 signalling.

Analysis of the deduced protein sequence of PIEZO using the Conserved Domain Database (CDD)^33^ revealed the presence of a unique PIEZO motif (amino acids 1235–1459) and R-Ras-binding domain (amino acids 2111–2519) at the C terminus. R-Ras is a member of the Ras family and can interact with Raf-1^34^, which activates the MEK and ERK1/2 pathways. Therefore, as R-Ras at the C terminus of PIEZO1 may be involved in the activation of MAPK, we tested the effects of the Ras inhibitor FTS on *BMP2* expression. FTS clearly inhibited *BMP2* expression by both 0.01 MPa HP loading (Fig. 5a) and Yoda1 treatment (Fig. 5b). Furthermore, to further confirm whether PIEZO1-Ras connection play an important role for BMP2 expression, *PIEZO1* siRNA transfected cells were examined in the presence of FTS. As the results, those were no significant difference of *BMP2* expression under FTS treatment between *PIEZO1* siRNA transfected cell and control siRNA transfected cells in the condition of 0.01 MPa HP loading (Fig. 5c) and Yoda1 treatment (Fig. 5d). These results suggested that Ras bindings at the C-terminus of PIEZO1 played an important role in the activation of MAPK and in the expression of BMP2.

### Piezo1 is essential for the caudal fin ray development *in vivo*

**Figure 6:**
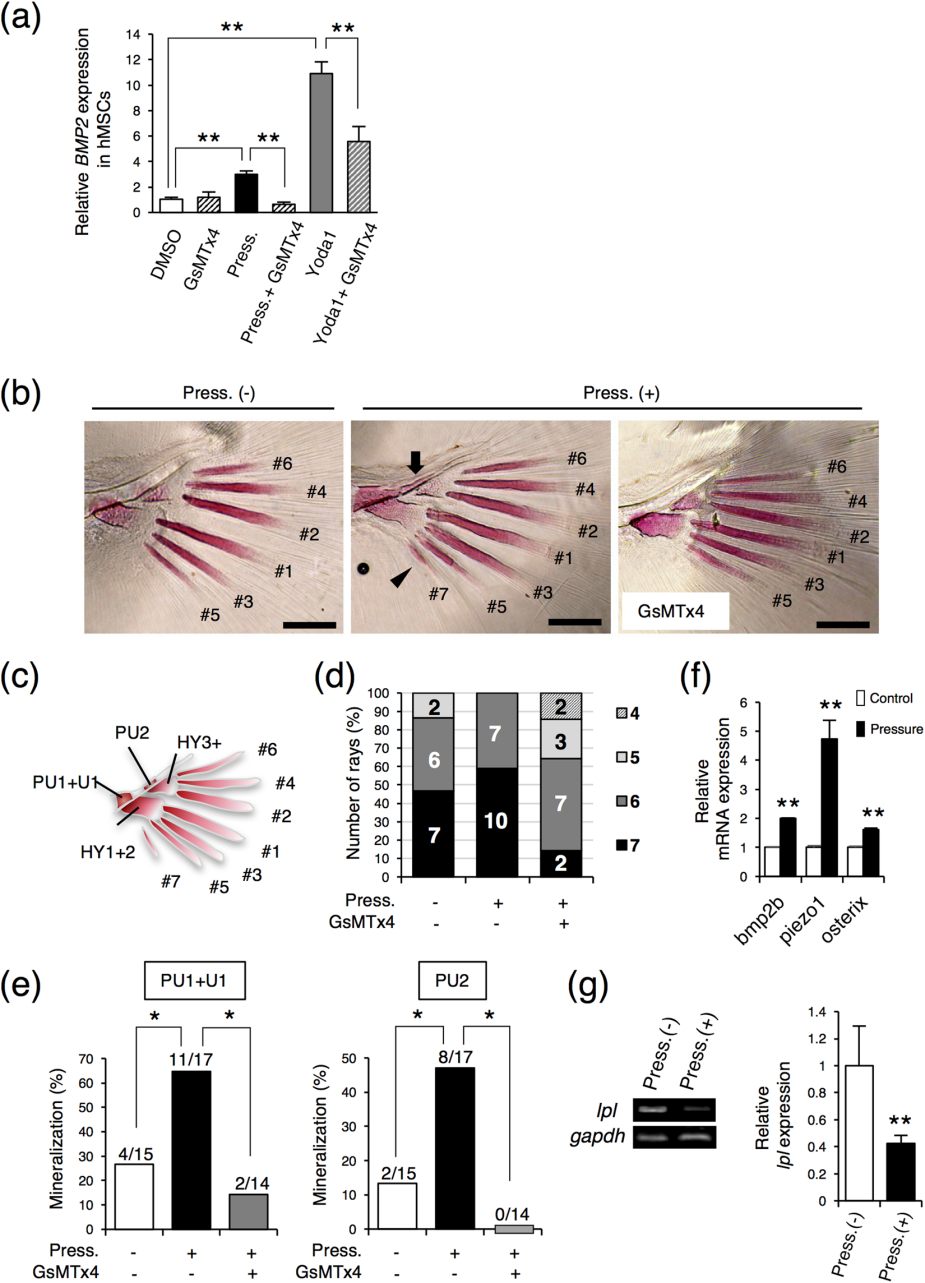
Piezo1 is involved in caudal fin ray development in medaka. (**a**) *BMP2* expression in GsMTx4 treated hMSCs. hMSCs were cultured with DMSO as a control or 10 μM GsMTx4 for 24 h under the condition of 0.01 MPa HP loading or in the presence of 5 μM Yoda1. Then, total RNA was then extracted from the cells and analysed by real-time RT-PCR. (**b**) Alizarin Red staining of the caudal fin ray. After hatching, medaka larvae were incubated at 0 or 0.02 MPa HP loading with or without 10 μM GsMTx4 (a piezo1 inhibitor) in a HP chamber for 3 days, and Alizarin Red staining was performed. Fin rays are formed in order from 1 to 7. The arrow and arrowhead indicates the second the preural centrum (PU2) and the seventh fin ray, respectively. Scale bar: 100 μm. (**c**) Schematic diagrams of the caudal fin ray. PU1 + U1, first preural centrum plus first ural centrum; PU2, second preural centrum; HY1 + 2, lower hypural plate; HY3+, upper hypural plate. (**d**) Number of caudal fin rays. Alizarin Red-positive staining of caudal fin rays was observed using a stereo microscope. (**e**) Number of Alizarin Red-positive PU1 + U1 and PU2. Alizarin Red-positive staining of PU1 + U1 and PU2 was observed using a stereo microscope, and were evaluated using the Fisher’s exact test (*p < 0.05). (**f**) Expression levels of *bmp2b*, *piezo1*, and *osterix* in the caudal fin. After hatching, the medaka larvae were incubated with or without 0.02 MPa HP loading in a HP chamber for 3 days. Then, total RNA was extracted from the caudal fin, and real-time RT-PCR for *bmp2b*, *piezo1*, and *osterix* was performed. (**g**) *lpl* expression was examined by RT-PCR. Statistical analysis of *lpl* expression was performed using ImageJ. Data were pooled from three independent experiments and error bars indicate standard deviations. Statistical analysis was performed using analysis of variance (**p < 0.01).

Finally, to confirm our hypothesis, medaka was used as *in vivo* model because it can be reared in a pressure chamber with application of HP. And we used the piezo1 inhibitor GsMTx4^35^ for suppression experiments. Before conducting *in vivo* experiments, we tested whether GsMTx4 had equivalent effects to knocking down PIEZO1 by siRNA. hMSC cells were treated with 10 μM GsMTx4. And then, *BMP2* expression was examined under the condition of 0.01 MPa HP loading or Yoda1 treatment. GsMTx4 inhibited the expression of *BMP2* induced by 0.01 MPa HP loading or Yoda1 treatment (Fig. 6a), indicating that in BMP2 expression, GsMTx4 has similar effects to PIEZO1 siRNA.

After hatching, medaka larvae were incubated in an HP chamber, and the early stage of fin ray development was observed under HP loading with or without the piezo1 inhibitor GsMTx4. In 15 fish without HP loading, an average number of 6.3 fin rays was observed at 3 days post-hatching (Fig. 6b). In 17 fish with HP loading, an average number of 6.6 fin rays was observed; no fish having 5 fin rays were observed (Fig. 6b and d). In contrast, among 14 fish with HP loading with GxMTx4, an average number of 5.6 fin rays was observed, including two fish with 4 fin rays and three fish with 5 fin rays was observed (Fig. 6b and d). Furthermore, at 5 days of post-hatching, the ossification of the first preural centrum plus first ural centrum (PU1 + U1) and second preural centrum (PU2) was observed (Fig. 6c). In the control group, the mineralization rate of PU1 + U1 and PU2 were approximately 26.7% and 13.3%, respectively (Fig. 6e). In the HP loading group, the mineralization rates of PU1 + U1 and PU2 were approximately 64.7% and 47.1%, respectively (Fig. 6e) whereas in the HP loading with GxMTx4 group, the mineralization rates of PU1 + U1 was about 14.3%, and no fish with mineralized PU2 was observed (Fig. 6e). These results indicated that HP loading promoted initial fin ray formation, and that piezo1 played a crucial role in fin ray development. In addition, real time-RT-PCR revealed that HP loading increased the expression of *bmp2* and *osterix* (Fig. 6f). Moreover, *piezo1* expression was enhanced by the HP loading (Fig. 6f) and HP loading significantly reduced the expression of the adipogenic marker *lpl* (Fig. 6g). Thus, HP promoted caudal fin ray development and might inhibit adipogenic cell differentiation, suggesting that the mechanosensing receptor piezo1 may also function in osteoblast differentiation in medaka fish.

## Discussion

In this study, we assessed how mechanical stimuli regulate the cell fate determination of MSCs. Extracellular environments are dynamic and complex properties that play a crucial role in the behaviors and functions of living cells. Among the factors affecting the extracellular environments, mechanical stimulation represents a principal component that influences growth during the foetal development process^36^; thus, mechanical forces also control cell fate specification and differentiation in all cells.

Indeed, all eukaryotic cells are mechanosensitive and are subjected to various mechanical forces, such as gravity, tension, compression and shear. Cells and tissues are surrounded by extracellular fluids, various mechanical stimuli are considered to be transmitted to them via extracellular fluids. Therefore, we focused on the effect of HP on MSCs. In the living body, intraocular pressure is typically 10–20 mmHg^37^, intracranial pressure is normally 7–15 mmHg^38^, intrauterine pressure is 40–50 mmHg under normal labour contractions^39^, and intracystic pressure of odontogenic jaw cysts is over 80 mmHg^40^. Normal blood pressure is in the range of 70–120 mmHg, and a systolic pressure of above 180 mmHg usually indicates a hypertensive crisis. Thus, the most suitable pressure for determining cell fate in MSCs is expected to be 0–200 mmHg; to this end, we created an original culture chamber to load HP in the range of 0–0.03 MPa (0–225 mmHg). We found that in MSC lines, the expressions levels of osteogenesis-related factors, such as *BMP2*, *RUNX2*, *OSX*, and *ALP*, were strongly upregulated in a force-dependent manner up to 0.01 MPa without the addition of osteogenic supplements. This suggested that 0.01 MPa HP was the most suitable for induction of osteoblast differentiation from MSCs; moreover, there may be optimum pressure conditions for differentiation of MSCs into different cell types.

In the current study, we found that exposure to continuous optimized HP could promote osteoblast differentiation of MSC lines more rapidly than normal induction culture. Mineralized nodule formation in osteogenic induction culture of MSC lines is usually observed in 3–4 weeks; however, in this study, mineralized nodule formation was dramatically increased with loading of 0.01 MPa continuous HP. These results suggested that continuous 0.01 MPa HP was suitable for induction of osteoblast differentiation from MSCs. However, further studies are needed to determine the effects of intermittent versus continuous HP.

We found, for the first time, that PIEZO1 plays an important role in the *BMP2* expression in MSCs. BMP2 is an important growth factor in osteoblast differentiation from MSCs^11^. However, the mechanism for controlling BMP2 expression in MSCs has not been cleared. Here, we found that HP significantly induced *BMP2* expression in hMSCs and MSC lines and that among mechanosensing receptors, *PIEZO1* was preferentially expressed in those cells. Downregulation of endogenous *PIEZO1* by siRNA resulted in substantial inhibition of *BMP2* expression and osteogenic differentiation. In previous studies, RUNX2 expression was found to be upregulated by mechanical stimuli including stretch^41^, pulsed ultrasound^42^ and HP^43^ during the osteogenic differentiation of MSCs. We also found that HP induced both *BMP2* and *RUNX2* expression in MSC lines. However, *BMP2*, but not *RUNX2*, was induced by PIEZO1 activator Yoda1. Conversely, PIEZO1 inhibitor GsMTx4 inhibited HP-induced *BMP2* expression. These results suggest that PIEZO1 plays a major role in BMP2 expression in MSCs. BMP2 expression may be the first step in cell fate determination process of MSCs. Although previous studies demonstrated that each activator and inhibitor tested on the functional specificities to PIEZO1^32,35^, we didn’t show any direct activities of PIEZO1 in this study. Thus, we need further studies that whether PIEZO1 activity is really involved in this process and PIEZO1 induction is really due to the activity of PIEZO1 itself or associated with the activity of other mechanosensing receptor, but it has been also reported that in neural stem cells, Piezo1 may play an important role for mechanosensitive lineage choice^27^. They also reported that PIEZO1, but not PIEZO2, was strongly expressed in adult hMSCs^27^. Collectively, these data suggest that PIEZO1 plays a major role for mechanosensitive cell fate determination in multipotent stem cells.

TRPV4 is another ubiquitously expressed mechanosensitive ion channel that plays a role in responses to a broad range of processes, from osmoregulation to thermosensing^44^. Suzuki *et al*.^45^ indicated that the expression of TRPV4 was enhanced upon differentiation of osteoblasts in culture. In current study, 0.01 MPa HP induced *PIEZO1* but not *TRPV4* expression. Furthermore, inhibition of *PIEZO1* expression by siRNA did not affect endogenous *TRPV4* expression; however, under these conditions, osteoblast differentiation from MSC lines was suppressed by *PIEZO1* siRNA. These results suggested that in MSCs, PIEZO1 served as a mechanosensing receptor that regulated initial osteoblast differentiation. Notably, in MC3T3-E1 cells, the expression of *Piezo1* increased in a manner similar to the expression of *ALP* up to day 3 of differentiation induction into osteoblasts (Fig. 2g). However, from day 3 to day 7, *ALP* expression increased by approximately 3.68-fold, whereas *Piezo1* levels increased only by about 1.03-fold (Fig. 2g). Thus, PIEZO1 may have important roles during the initial rather than the terminal differentiation stage of osteoblast differentiation. Recently, Piezo1 was found to be involved in the regulation of cell numbers and to function to promote rapid epithelial cell division under mechanical stress^46^, consistent with requirement during the early phase of osteoblast differentiation. In addition, although both TRPV4 and PIEZO1 share some properties as mechanical receptors, they are functionally quite different^47,48^, suggesting that PIEZO1 and TRPV4 may play distinct roles in osteogenic differentiation.

Activation of the ERK1/2 signaling pathway is required for the signal transduction of mechanical force in many cell types^49^. Thus, the ERK1/2 pathway may be an important signaling pathway in cellular mechanotransduction. In rat incisor dental pulp cells, ERK1/2 was activated in 24 hours after treatment with low-intensity pulsed ultrasound (LIPUS) but ruthenium red (RR), a Piezo ion channel blocker, inhibited the ERK1/2 activation^50^. Here, we also demonstrated that HP loading activated ERK1/2 and p38, but not JNK. In addition, Yoda1 also activated both ERK1/2 and p38, but not JNK. The MEK inhibitor U0126 and the p38 inhibitor SB203580 suppressed HP- and Yoda1-induced *BMP2* expression. These results suggested that both ERK1/2 and p38 might act as signalling molecules in PIEZO1-BMP2 expression. Furthermore, we found that FTS, an inhibitor of Ras, also inhibited HP- and Yoda1-induced *BMP2* expression. These results suggest that Ras signalling at the C terminus of PIEZO1 may play a role in modulating BMP2 expression.

The Smad signalling pathway has fundamental roles in osteoblast differentiation in response to BMP ligands^51^. However, we found that the Smad phosphorylation was not induced by HP loading within 30 min. Because immediate early signaling events downstream of the BMP receptors occur within 30 min of BMP2 stimulation, cellular signalling associated with HP may not be directly associated with Smad signalling. However, Kopf J *et al*.^52^ suggested that the induction of Smad phosphorylation by BMP2 was enhanced at 15 and 30 min after mechanical loading. These results suggested that the induction of endogenous BMP2 expression by HP is a crucial role for acceleration of osteoblast differentiation from MSCs under the HP loading. In addition, our findings indicated the HP and Yoda1 induced the phosphorylations of ERK1/2 and p38, resulting in activation of osteoblast differentiation and suppression of adipogenic differentiation, consistent with previous findings that the commitment of hMSCs into osteogenic or adipogenic lineages was governed by the activation or inhibition of ERK, respectively^53^. Furthermore, several studies have suggested that p38 activity blocks adipocyte differentiation^54,55^. However, other reports have shown that both ERK1/2 and p38 are essential for adipocyte differentiation^56^. In a variety of cell types, MAPK signalling pathways play important roles in many essential and complex cellular events, such as proliferation and differentiation. MAPK signalling is also involved in adipogenesis, displaying both positive and negative effects depending on the stage of differentiation^57^. Our findings suggested that the early activation signals of ERK1/2 and p38, induced by optimum pressure for osteoblast differentiation of MSCs, negatively regulated their adipocyte differentiation. However, although BMP2 is an important factor in osteoblast differentiation, it is known that in rodent cells and human cells, there is a difference in response to BMP2 due to complicated mechanisms of intracellular signals^15^. In this study, we demonstrated for the first time that PIEZO1 is crucial for BMP2 expression, but we will need more detailed analysis on intracellular signalling pathways in HP response and subsequent osteoblast differentiation.

In conclusion, the cell fate decisions of MSCs are highly regulated according to cellular conditions. Moreover, mechanical stress, particularly HP through extracellular fluid, was found to be a principal anabolic factor affecting osteoblasts differentiation. We suggested that PIEZO1 acts as a receptor for HP and functions at the branch points of cell fate decisions of MSCs by regulating BMP2 expression. We believe that these findings provide critical insight into the molecular mechanisms controlling the balance between osteoblastogenesis and adipogenesis and the cellular response to mechanical force, which may facilitate the development of novel therapeutic strategies for the treatment of skeletal diseases.

## Material and Methods

### Reagents

SB203580 (p38 inhibitor), and a kit for enzyme activity staining of ALP were purchased from WAKO (Osaka, Japan). The Alizarin Red staining kit was obtained from PG Research (Tokyo, Japan). The Oil Red O stainning kit was purchased from Sigma-Aldorich (MO, USA). GsMTx4 and Yoda1 were purchased from Abcam (Tokyo, Japan) and Tocris Bioscience (Bristol, UK), respectively. Recombinant murine noggin was obtained from PeproTech (NJ, USA). U0126 was purchased from Cell Signaling (MA, USA).

### Cell and Cell culture

The UE7T-13 cells, a human bone marrow-derived MSC line infected with retroviruses expressing papillomavirus E7 and hTERT, were purchased from the Health Science Research Resources Bank (JCRB1154; Japan Health Sciences Foundation, Tokyo, Japan). The SDP11 cells, a human dental pulp-derived MSC line transfected with the *pBABE-neo-hTERT* plasmid, were recently established from the crown and root of healthy human deciduous teeth^58,59^. The primary human MSCs originated from a single human bone marrow (BMSCs) were obtained from Lonza (PT-2501). The cells were maintained with α-modified minimum essential medium (α-MEM; Gibco-BRL, Gaithersburg, MD, USA) containing 10% fetal bovine serum (FBS; Gibco-BRL) at 37 °C in a humidified atmosphere of 5% CO_2_ and media were replaced every 2 days. The time course of cell cultures for osteoblast or adipocyte inductions from MSC lines were performed as previously described with some modifications^60,61^. For osteogenic differentiation, cells were seeded at 3.0 × 10^4^/ well and cultured in α-MEM supplemented with 10% FBS, 10 mM glycerophosphate, 150 μg/mL ascorbic acid, and 10^−8^ M dexamethasone. Osteogenesis was determined by ALP activity and Alizarin Red S staining for calcium deposition according to the manufacturer’s protocol. ALP- and alizarin red S-positive areas were quantified by NIH-ImageJ. For adipogenic differentiation, cells were seeded at 1.2 × 10^6^ cells per well in 60-mm dishes and cultured in α-MEM supplemented with 10% FBS, 0.5 mM isobutylmethylxanthine, 0.5 µM hydrocortisone, 60 µM indomethacin, and 10 µg/mL insulin. Adipogenesis was determined by Oil Red O staining according to the manufacturer’s protocol. Oil Red O positive staining was quantified by NIH-ImageJ. The human osteosarcoma cell lines (Saos-2, HuO9, and MG63) and a clonal murine calvarial MC3T3-E1 cells were used as a model for osteoblasts. Saos-2 and MG63 were maintained in standard growth medium composed of DMEM. HuO9 and MC3T3-E1 were maintained with RPMI1640 and α-MEM, respectively. Each medium was supplemented with 10% FBS and 1% pen/strep. Osteogenesis induction in MC3T3-E1 cells was cultured under the same conditions as the osteoinductive conditions described above. The study protocol was approved by the Ethics Committee of Tokushima University Hospital (no. 1799), Tokushima, Japan.

### Medaka fish

Adult Japanese medaka (Himedaka, *Oryzias latipes*) were maintained at a temperature of 25 °C under a 14-h/10-h light/dark cycle in the laboratory. Eggs were obtained from random crossing, and embryos were incubated at 30 °C. After hatching, the larvae were incubated at 25 °C. The experimental procedures were conducted in accordance with the guidelines for animal experimentation of Tokushima University.

### Hydrostatic pressure experiment

We designed a custom-made pressure chamber fabricated for hydrostatic pressure experiments. In this study, the atmospheric pressure was regarded as the zero reference and the hydrostatic pressure was expressed as the gage pressure.

The cells or medaka were exposed to the mechanical stimulus through the medium or water by increasing the pressure of the gaseous phase of 5% concentration of CO_2_ gas or atmospheric air. The temperature was controlled by placing the pressure chamber in a thermostatic oven (Fig. 1c). Cells seeded on 35-mm dishes were placed in a 150-mL beaker and incubated with 100 mL medium, 5% CO_2_ and 37 °C in a pressure chamber under HP loading. Control cells were cultured under the same conditions without HP. Newly hatched medaka larvae were transferred into 24-well plates with 500 µL water and placed into a pressure chamber. Medaka larvae were reared at 25 °C under a 100% atmosphere with or without HP.

### RT-PCR and quantitative RT-PCR

Total RNA was extracted with TRIzol reagent (Invitrogen, Carlsbad, CA, USA) according to the manufacturer’s protocol. First-strand cDNA was synthesized from 2 μg of total RNA with the PrimeScript RT Master Mix (Perfect Real Time; Takara). RT-PCR was performed with KOD Plus (TOYOBO). cDNA was amplified by initial denaturation at 95 °C for 3 min; 25 cycles of 95 °C for 30 s, 60 °C for 30 s, and 72 °C for 30 s; and a final elongation step at 72 °C for 5 min. PCR products were separated on 2% agarose gels. Quantitative PCR was carried out using PCR SYBR Premix Ex Taq II (Takara) and a Thermal Cycler Dice real-time system (Takara). The conditions of amplification were as follows: 10 s at 95 °C, followed by 40 cycles of 95 °C for 5 s and 60 °C for 30 s, with a final 5 s at 95 °C and 30 s at 60 °C. Gene expression was normalized to the housekeeping gene, glyceraldehyde-3-phosphate dehydrogenase (*GAPDH*). The reactions were run in triplicate and repeated three times, and the results were combined to generate the graphs. We used the primers listed in Supplementary Table S1.

### Western blotting

Cells were washed three times with phosphate-buffered saline containing 1 mM sodium vanadate (Na_3_VO_4_) and then were then lysed in ice-cold Sigma CellLytic™ M reagent supplemented with Complete Mini Protease Inhibitor Cocktail tablets (Roche) for 10 min. Lysed cells were centrifuged at 12,000 rpm for 5 min at 4 °C, and the protein concentration of each sample was measured with BCA assay reagent (Thermo Fisher Scientific). The samples were denatured in LDS sample buffer with sample reducing agent (Invitrogen) and loaded onto a NuPAGE™ 4–12% Bis-Tris Protein Gels (Invitrogen), with 10 µg of lysate protein being applied to each lane. After SDS-PAGE, the proteins were transferred onto a polyvinylidene disfluoride membranes and immunoblotted with antibodies targeting PIEZO1 (Novus), p44/42, phospho-p44/42, p38 MAPK, phospho-p38 MAPK, SAPK/JNK, phospho-SAPK/JNK, Smad5, phospho-Smad1/5/8, and β-actin (Cell Signaling). Finally, membranes were visualized using an ECL kit (GE Healthcare, Chicago, IL).

### siRNA experiments

siRNA transfections were performed using Lipofectamine™ RNAiMAX Transfection Reagent (Invtitrogen) following the manufacturer’s protocol. Briefly, cells were plated at 1 × 10^5^ cells/well in 24 well plates and the transfection complex (containing 1.5 μL Lipofectamine™ RNAiMAX and siRNAs) was added directly to the medium. RNA and protein samples were isolated from cells 48 h post-transfection. The following siRNAs were used: ON-TARGETplus Non-targeting siRNA pool (D-001810-1005, Dharmacon, Lafayette, CO), Set of 4: ON-TARGETplus PIEZO1 siRNAs (LQ-020870-03-0002, Dharmacon).

### Immunohistochemistry

The cells were fixed with 4% paraformaldehyde at room temperature for 5 min. Immunohistochemistry was performed on sections that were incubated with Universal Blocking Reagent (Biogenex, San Ramon, CA) for 6 min at room temperature before incubation with the primary antibody. The primary antibody was detected by Alexa Fluor 594-congugated AffiniPure Goat Anti-Rabbit IgG (H + L) (Invitrogen). An Olympus BX50 microscope (Tokyo, Japan) was used for immunofluorescence image analysis.

### Statistical Analysis

For the analyses represented in Figs 1–4, and Fig. 5e and g, data were pooled from three independent experiments, and error bars indicate standard deviations. Statistical analysis was performed using analysis of variance (*p < 0.05, **p < 0.01,). For the analysis represented in Fig. 5d, data were evaluated using the Fisher’s exact test (*p < 0.05).

## Electronic supplementary material

Supplementary Dataset 1
