# Supplementary material for: Piezo type mechanosensitive ion channel component 1 functions as a regulator of the cell fate determination of mesenchymal stem cells
Source: Sci Rep. 2017 Dec 18;7:17696. doi: 10.1038/s41598-017-18089-0 (PMC5735093; doi:10.1038/s41598-017-18089-0)
Supplement: Supplementary file 1 — Supplementary Dataset 1 [file 41598_2017_18089_MOESM1_ESM.pdf]

Piezo type mechanosensitive ion channel component 1 functions as a regulator of the cell fate determination of mesenchymal stem cells

Asuna Sugimoto[1], Aya Miyazaki[1], Keita Kawarabayashi[1], Masayuki Shono[2], Yuki Akazawa[1], Tomokazu Hasegawa[1], Kimiko Ueda[1], Takamasa Kitamura[1], Keigo Yoshizaki[3], Satoshi Fukumoto[4], Tsutomu Iwamoto[1*]

Supplementary Figure S1

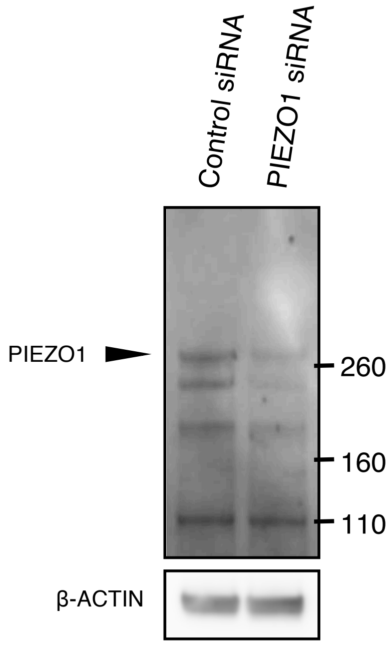

Supplemental Figure S1. Reduced expression of endogenous PIEZO1.
UE7T-13 cells were transfected *PIEZO1* siRNA or control siRNA. Protein was prepared from cells after 24 h of culture and analyzed through western blotting using PIEZO1 antibody. Beta-actin was used as a loading control.

Supplementary Figure S2

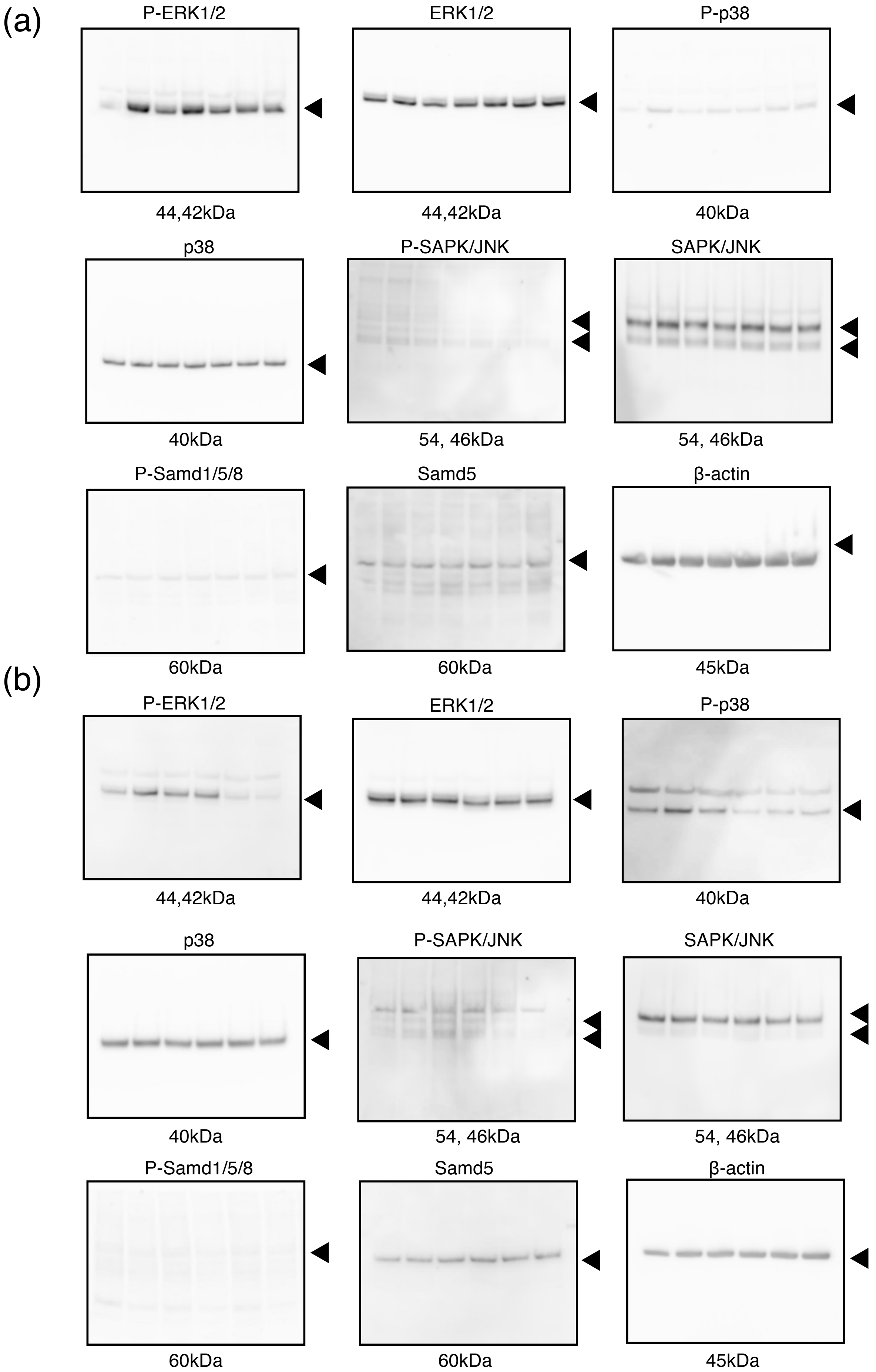

Supplementary Figure S2

(c)

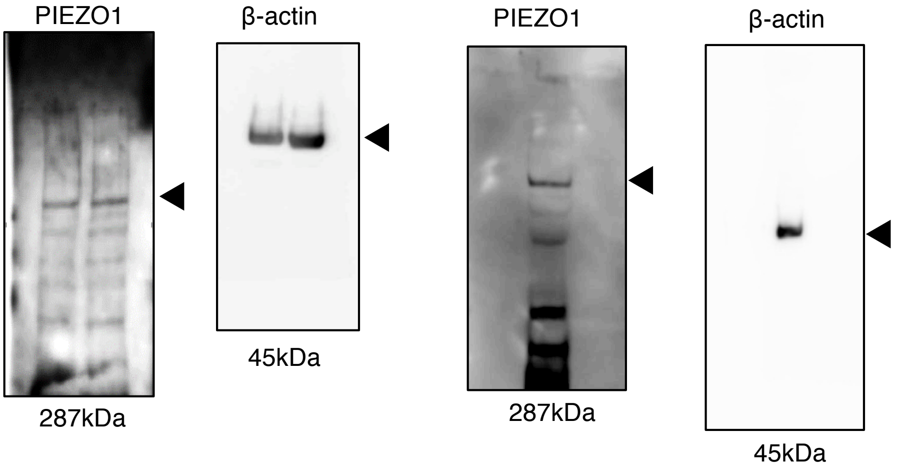

(d)

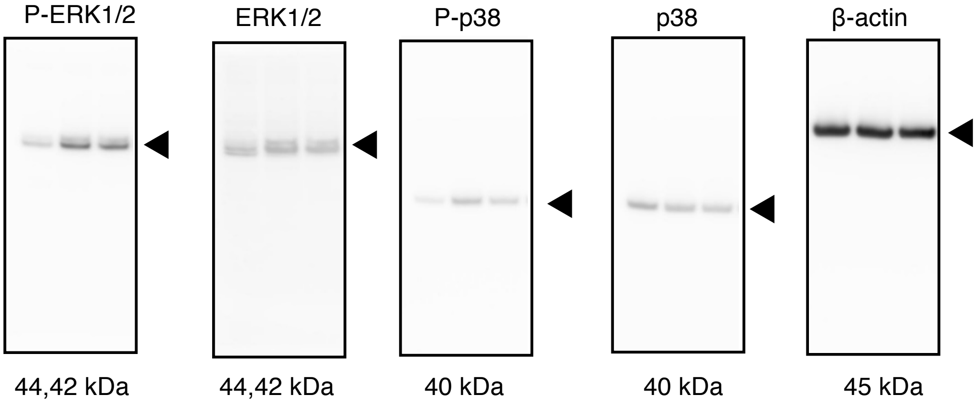

(e)

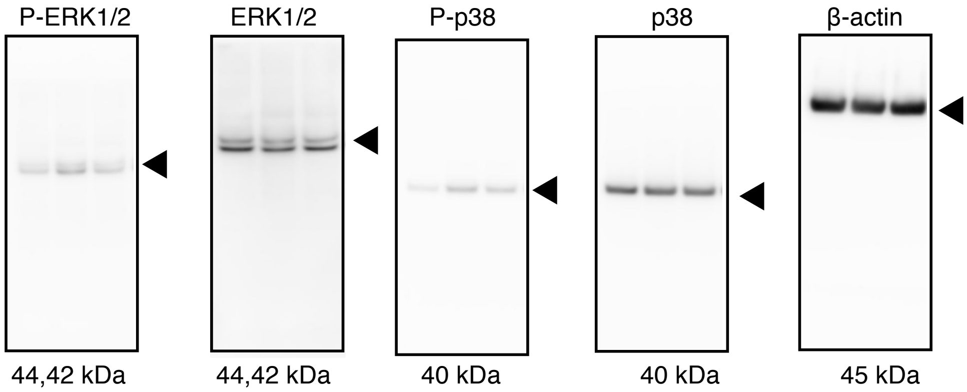

Supplementary Figure S2

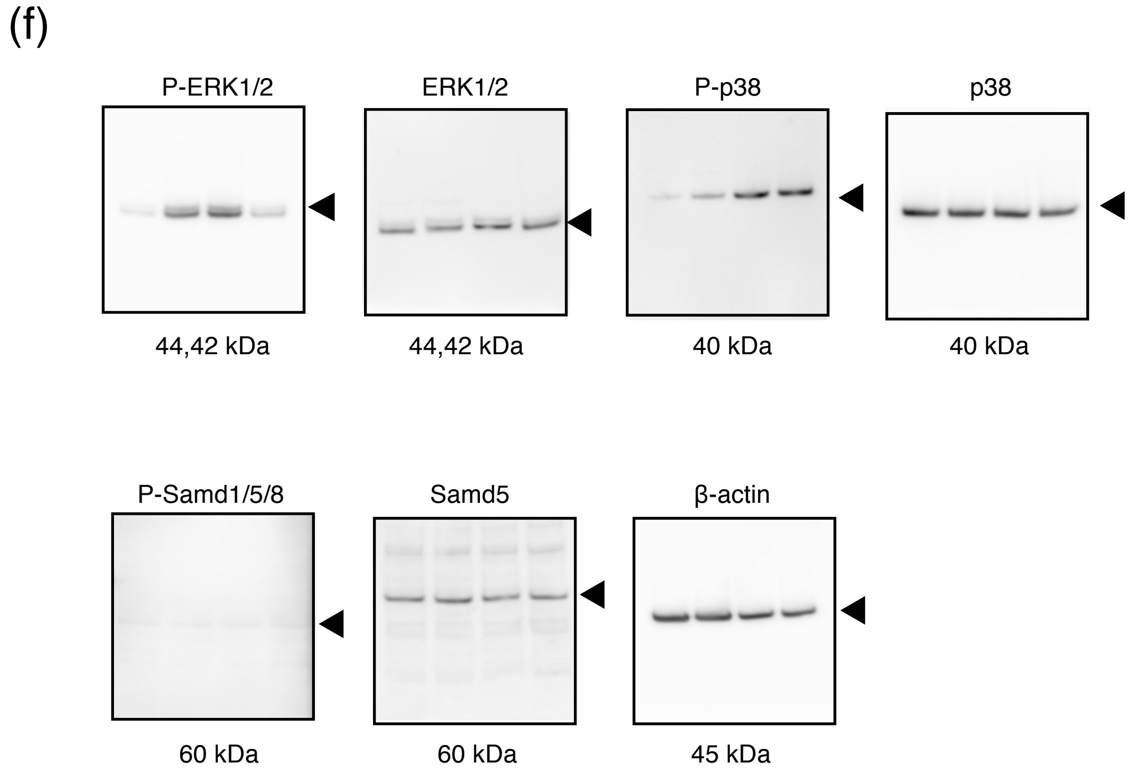

Supplemental Figure S2. Full-length pictures of western blotting.
(a, UE7T-13) and (b, SDP11) are full-length blots for Fig.2(a). (c) is full-length blots for Fig.2(c). (d, UE7T-13) and (e, MG63) are full-length blots for Fig.3(c). (f) is full-length blots for Fig.4(b). Arrow heads represent each band as expected size.

Supplementary Figure S3

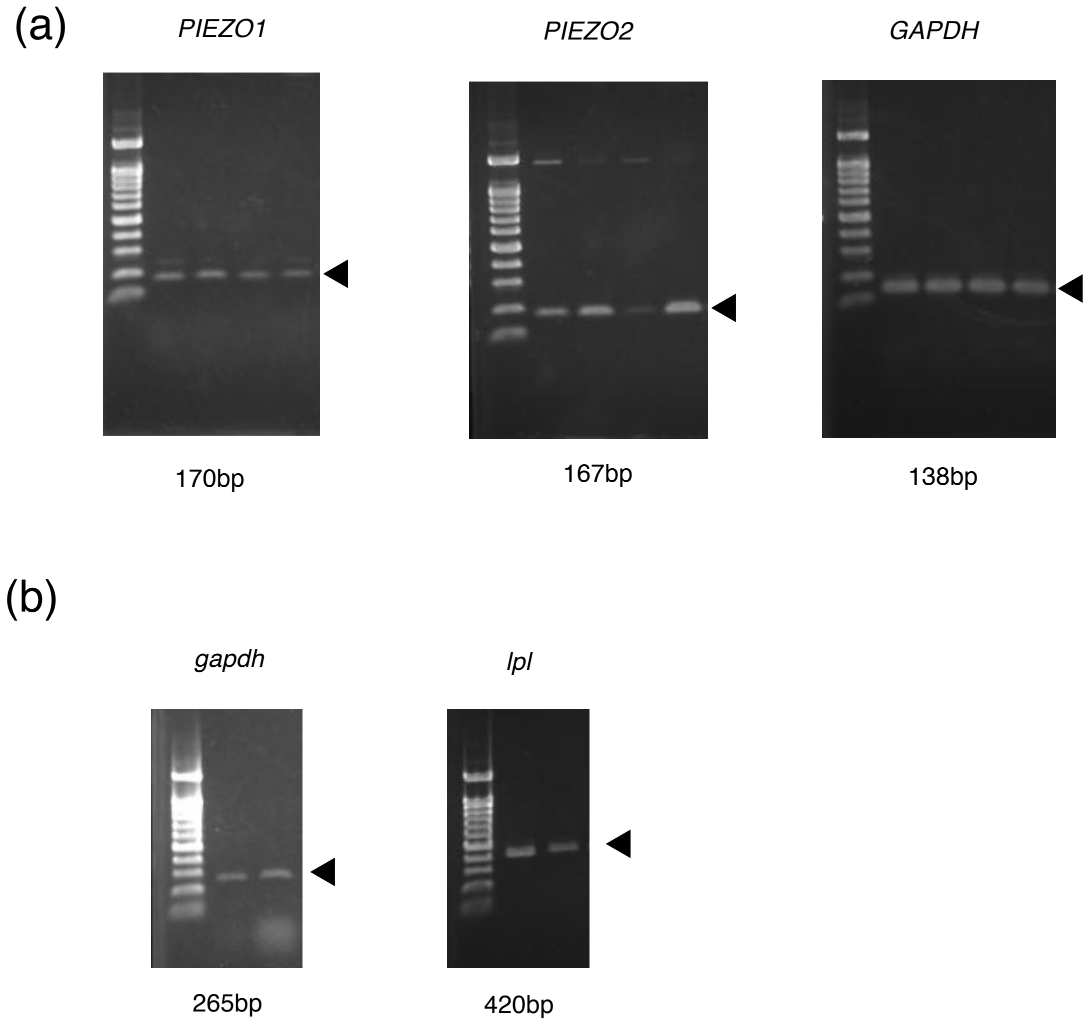

Supplemental Figure S3. Full-length pictures of RT-PCR .
Full-length RT-PCR for Fig.2f (a) and Fig.6g (b). Arrow heads represent each band as expected size.

Supplementary Figure S4

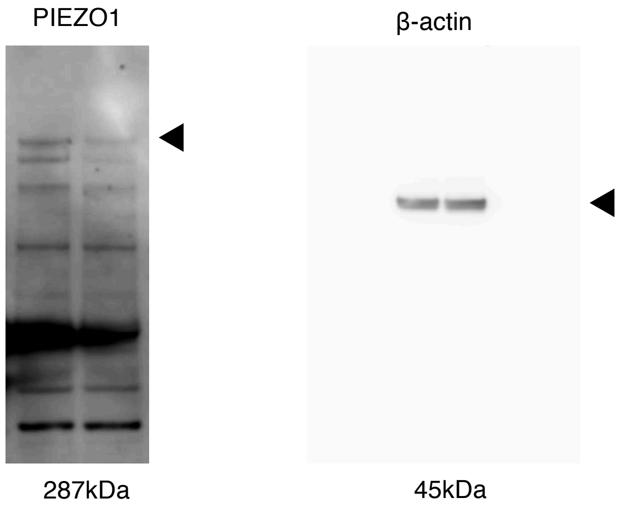

Supplemental Figure S4. Full-length pictures of western blotting in Supplemental Fig. S1.
Full-length blots for Supplemental Fig.S1. Arrow heads represent each band as expected size.

Supplementary Table S1

Used primers for PCR

Human primers		Sequence	Product size
BMP2	forward	5’-CCCAGCGTGAAAAGAGAGAC-3’	222
	reverse	5’-GGAAGCAGCAACGCTAGAAG-3’	
RUNX2	forward	5’-TTACCCCTCCTACCTGAGCCAG-3’	289
	reverse	5’-GGTGTGGTAGTGAGTGGTGG-3’	
OSX	forward	5’-TGCTTGAGGAGGAAGTTCAC-3’	148
	reverse	5’-AGGTCACTGCCCACAGAGTA-3’	
ALP	forward	5'-GGACCATTCCCACGTCTTCAC-3’	137
	reverse	5’-CCTTGTAGCCAGGCCCATTG-3’	
COL1A1	forward	5’-ACTGGTGAGACCTGCGTGTAC-3’	435
	reverse	5’-ATGGGCAGGCGGGAGGTCTTG-3’	
LPL	forward	5’-TGTGGTGGACTGGCTGTCA-3’	73
	reverse	5’-CTGTCCCACCAGTTTGGTGTAG-3’	
PIEZO1	forward	5’-CAGGCCTATGAGGAGCTGTC-3’	170
	reverse	5’-TTGTAGAGCTCCCGCTTCAT-3’	
PIEZO2	forward	5’-GCCCAACAAAGCCAGTTGAA-3’	167
	reverse	5’-GGGCTGATGGTCCACAAAGA-3’	
TRPV4	forward	5’-GACGGGGACCTATAGCATCA-3’	228
	reverse	5’-AACAGGTCCAGGAGGAAGGT-3’	
ASIC3	forward	5’-ATGGCGCAACTCTATGCCCGT-3’	202
	reverse	5’-TGATGTCCAGCCCATTGCCCA-3’	
GAPDH	forward	5’-GCACCGTCAAGGCTGAGAAC-3’	138
	reverse	5’-TGGTGAAGACGCCAGTGGA-3’	
Mouse primers		Sequence	Product size
Alp	forward	5’-TGATCATTCCCACGTTTTCAC-3’	202
	reverse	5’-CTGGGCCTGGTAGTTGTTG-3’	
Piezo1	forward	5'-CACTCTGCAGCCACAGACAT-3'	463
	reverse	5'-CACACATCCAGTTGGACAGG-3'	
Gapdh	forward	5'-GGAGCGAGACCCCACTAACATC-3'	181
	reverse	5'-CTCGTGGTTCACACCCATCAC-3'	
Medaka primers		Sequence	Product size
bmp2b	forward	5’-AGCATGGGAAAACACGCGGAGA-3’	206
	reverse	5’-TTTGCGCCCCCTTTCACCTGA-3’	
piezo1	forward	5’-TGCTTTGTGTGGGCATTCCGC-3’	300
	reverse	5’-TGAAGTTCGGCGCAGGGTTGA-3’	
osterix	forward	5’-TGTTTCTCAGGGATGGCCGCA-3’	153
	reverse	5’-TTGAGTGGTGCTGCCGGTCTT-3	
lpl	forward	5'-CACGGGGAGACAGAGGAC-3'	420
	reverse	5'-CGCCGTGTCCTTTCTTTCTTT-3'	
gapdh	forward	5'-AGCTGCCAAGGCTGTGGGAAA-3'	265
	reverse	5'-TGCCGGCACCAGCATCAAAGA-3'	
